# Blood donor eligibility after COVID-19 vaccination: the current state of recommendations

**DOI:** 10.11604/pamj.2021.40.207.29119

**Published:** 2021-12-06

**Authors:** Sabah Bouhou, Khadija Lahjouji, Azlarab Masrar

**Affiliations:** 1 National Centre for Blood Transfusion and Hematology, Rabat, Morocco,; 2 Hematology Laboratory, Faculty of Medicine and Pharmacy, Mohammed V University, Rabat, Morocco,; 3 Central Hematology Laboratory, Ibn Sina University Hospital Centre, Rabat, Morocco

**Keywords:** Vaccination, eligibility, blood donation, COVID-19, recommendations, blood Safety

## Abstract

The medical screening practices for blood donor eligibility after vaccination against several conventional infectious agents are well known to blood transfusion centres worldwide. Classically, blood donations are contraindicated for four weeks after vaccination with a live attenuated virus vaccine. For other types of vaccines, no postponement is necessary as there is no proven infectious risk. But for the COVID-19 vaccine, besides the classic platforms for the vaccine, two new types of vaccine platforms have been developed and based on viral vectors vaccines and vaccines from viral genetic material. Thus, blood establishments could be worried that these COVID-19 vaccine production platforms can pose any particular safety concerns for recipients of blood or blood products from vaccinated subjects. Our work aims to provide professionals in blood transfusion centres with all the available information to clarify any confusion relating to the need or not to defer donors who have received a COVID-19 vaccination. So in this paper, we provide an international review of the data available to date about the recommendations of international scientific societies about blood donation after COVID-19 vaccination, all types of COVID-19 vaccines included. We also present some experiences of blood transfusion establishments worldwide concerning this topic and among them our institution, the Moroccan National Centre for Blood Transfusion and Haematology.

## Introduction

Since the beginning of the COVID-19 pandemic, blood establishments worldwide have continuously updated the eligibility criteria for donating blood based on the evolution of the international epidemiological situation with the SARS-CoV-2 virus and according to the development of scientific knowledge concerning this new coronavirus. With the start of COVID-19 vaccination campaigns, questions arise about the duration of exclusion (or the lack of exclusion) of blood donors who have received the COVID-19 vaccine. In this context, it´s essential to know that making an inappropriate decision to defer people vaccinated with the COVID-19 vaccine could exclude a large part of the population from blood donation for several weeks and possibly compromise self-sufficiency in labile blood products. The decision to defer a donor after COVID-19 vaccination should be based essentially on the reel risk of transmission of a potentially dangerous infectious agent to recipients of blood products [1].

The medical screening practices for blood donor eligibility after vaccination against several conventional infectious agents are well known to blood transfusion centers worldwide. For classic infectious agents, blood donation is contraindicated four weeks after injection of a live attenuated vaccine. Other types of vaccines (inactivated vaccines, antigens, or protein subunits) do not entail any risk of infection and do not justify any postponement [1]. For the COVID-19 vaccine, according to the World Health Organization October 2020 list, nearly 200 candidate vaccines against SARS-CoV-2 are under development, using eight different technological platforms: live attenuated and inactivated vaccines, vaccines with a base of protein subunits, vaccines from viral genetic material (RNA, DNA), vaccines by a replicative or non-replicative vector, and vaccines with viral pseudo-particles “virus-like particles” [2]. Deoxyribonucleic acid (DNA) and RNA platforms have never been the source of vaccines yet marketed in humans [1,2]. Thus, through our review, we seek to give clear answers to the fact that all these technical platforms for producing the COVID-19 vaccine may require a change in the medical selection practices of blood donation at the level of blood transfusion establishments.

## Methods

From 1^st^ December 2020 to 31^st^ April 2021, we searched data about the eligibility criteria for blood donation after receiving the COVID-19 vaccine. For this, we consulted several websites such as PubMed Central, Hinari, Web of Sciences, Google Scholar, Google, World Health Organization (WHO) website, European Centre of Disease Control (ECDC) website, Food and Drug Administration (FDA) website, International Society of Blood Transfusion (ISBT) website, American Association of Blood Banks (AABB) website, the Centre for Disease Control (CDC) website, the High Council for Public Health (HCPH) website. We also consulted websites of blood establishments worldwide like American Red Cross, Australian Red Cross, Scottish National Blood Transfusion Service, Canadian blood services. We used several key search terms such as blood donation after COVID-19 vaccine; eligibility criteria and COVID-19 vaccination; the impact of COVID-19 vaccination on blood donation; blood donor´s deferral after COVD-19 vaccine; technical platforms of production of COVID-19 vaccines; COVID-19 vaccines production; COVID-19 vaccines and blood donation screening. All the data collected was analysed and synthesized into relevant information reported in our article.

## Current status of knowledge

**Brief overview of the new vaccine platforms used for the production of the COVID-19 vaccine:** in the context of emerging pathologies, it appeared desirable to have platforms that can easily be used to rapidly develop vaccines capable of inducing humoral and cellular responses without requiring large doses of vaccine [2]. Vaccines based on the use of gene fragments encoding proteins of interest, rather than whole viruses, or some of these proteins themselves, meet these criteria [1,2]. There are two types: viral vectors (replicative or no replicative) and nucleic acid vaccines, deoxyribonucleic acid and ribonucleic acid (DNA and RNA). These new platforms are used for the production of the COVID-19 vaccine [1,2]. The viral vectors vaccine production consists of using a virus that is not very pathogenic (adenovirus) or that has been made non-pathogenic (modified ankara virus (MAV), vesicular stomatitis virus (VSV)) [1,2]. They integrate into its genome the sequence encoding the protein of interest, a spike protein in the case of SARS-CoV-2 [1,2]. For the adenoviral non-replicative platforms, DNA viruses are made non-replicating. There are over 90 human adenovirus serotypes [1,2]. For SARS-CoV-2 vaccines, three adenoviruses are used: Ad5 and Ad26, which infect humans, and a chimpanzee adenovirus ChAdOx1 [1,2]. It´s expected that adenoviruses (Ad) acquired by natural infections can persist for years in the lymph nodes and tonsils. Adenoviruses vectors derived from human serotypes or chimpanzees lacking E1 appear to share some of these traits, staying at the inoculation site, in the liver, and in lymphatic tissues. This persistence is not associated with virus replication [1,2]. Like viral vectors, DNA and mRNA vaccines allow the production of antigens inside cells and, therefore, the supply of proteins with all the required post-translational modifications [1,2].

The first DNA vaccines were developed about 30 years ago. To produce these vaccines, they use a bacterial plasmid into which they insert a eukaryotic expression cassette encoding the vaccine antigen, which comprises a CMV promoter and a 3'-polyadenylation signal encoding the vaccine antigen. The plasmids are then amplified in *Escherichia coli* (E.coli) and therefore contain the elements promoting their production in this bacterium [1,2]. Deoxyribonucleic acid vaccines have the advantage of being relatively simple to produce, inducing a humoral and cellular response, and being very stable. Unfortunately, they are not very immunogenic, requiring several doses as a primary vaccination [1,2]. Ribonucleic acid manufacture is based on processes of a chemical and not biological nature, such as the vectors described above. Their large-scale production is greatly facilitated. This platform is non-infectious and with no integration capacity. There is no potential risk of infection or insertional mutagenesis [1,2]. At this stage of information, we know that for COVID-19, no DNA-based vaccine, live attenuated vaccine, or replicative vector vaccine has yet been marketed in the world [1]. The COVID-19 vaccines now available worldwide are not expected to pose any particular safety concerns for recipients of blood products from vaccinated subjects [1]. We presented in Table 1 an illustration of information on the characteristics of the COVID-19 vaccine platforms and COVID-19 vaccines, currently available worldwide [3]. But, what those international scientific societies think about the blood donation after COVID-19 vaccination, all types of vaccines included?

**Table 1:** illustration of information on the characteristics of the COVID-19 vaccine platforms and COVID-19 vaccines, currently available worldwide

Vaccine platform	Type of vaccine	Developers	Phase
Inactivated virus	Inactivated SARS-CoV-2 vaccine (vero cell)	Sinopharm+ China National biotec group Co+Wuhan institute of biological products	Phase 4
	Inactivated SARS-CoV-2 vaccine (Vero cell), vaccine name BBIBP -CorV	Sinopharm+ China National biotec group Co+Bejing institute of biological products	Phase 4
	CoronaVac; inactivated SARS-CoV-2 vaccine (Vero cell)	Sinovac research and development Co.ltd	Phase 4
	Whole-Virion inactivated SARS-CoV-2 vaccine (BBV152)	COVAXIN, Bharat biotech international limited,India	Phase 3
mRNA-based vaccine	mRNA-1273	Moderna+ National institute of allergy and infectious diseases(NIAID)	Phase 4
	BNT162b2(3LNP-mRNAs),also known as ''Comirnaty''	Pfizer/BioNTech+Fosun Pharma	Phase 4
Non-replicating viral vaccine	ChAdOx1-S- (AZD1222) Covishield Vaxzevria	AstraZeneca + University of Oxford	Phase 4
	Recombinant novel coronavirus vaccine (Adenovirus type 5 vector) /Ad5-nCoV	CanSino Biological Inc./Bejing institute of biotechnology	Phase 4
	Gam-COVID-Vac Adeno-based (rAd26-S+rAd5-S) Sputnik V COVID-19 vaccine	Gamaleya research institute; health Ministry of the Russian Federation	Phase 3
	Ad26.COV2.S	Janssen Pharmaceutical/Johnson & Johnson	Phase 4
Vaccine with protein subunit	SARS-CoV-2 rS/Matrix M1-Adjuvant (full length recombinant SARS-CoV-2 glycoprotein nanoparticle vaccine adjuvanted with matrix M) NVX-CoV2373	Novavax	Phase 3
Live attenuated vaccine	**-**02 candidate vaccines are in clinical development (01 in Phase 1- 01 in Phase 3) /WHO List -02 candidate vaccines are in pre-clinical development/WHO List Not yet marketed		
Replicative viral vaccine	-05 candidate vaccines are in clinical development /WHO List: 01 in Phase 01 in Phase 1/2 01 in Phase 2 01 in Phase 2/3 01 in Phase 3 **-**19 candidate vaccines are in pre-clinical development /WHO List Not yet marketed		
DNA-based vaccine	**-**15 candidate vaccines are in clinical development /WHO List: 07 in Phase 1 05 in Phase 1/2 02 in Phase 2/3 01 in Phase 3 **-**16 candidate vaccines are in pre-clinical development /WHO List Not yet marketed		

**Data from international scientific societies**: the European Centre for Disease Prevention and Control (ECDC), on its second technical update published on December 10^th^ 2020, stipulated that after vaccination with attenuated viruses (e.g. virus vector-based or live-attenuated virus vaccines), donors must be deferred for four weeks [4]. Individuals vaccinated with inactivated viruses or vaccines that do not contain live agents (i.e. mRNA and protein subunit vaccines) may be accepted as donors if they feel well [4]. If information about vaccine type is missing or the vaccination is experimental, a four-week deferral period should be applied [4]. Given that COVID-19 vaccines are newly developed, member states may consider taking a precautionary approach and deferring donors who develop symptoms directly after receiving a SARS-CoV-2 vaccine for up to seven days after symptoms have resolved [4]. In addition, donors need to comply with the same general requirements for donation as non-vaccinated donors [4]. A regulatory update of the American Association of Blood Banks (AABB) on November 20^th^ 2020, reported that the potential for a growing number of donor deferrals for investigational COVID-19 vaccines could pose another challenge to the blood supply [5]. In response to AABB, food and drug administration (FDA) stated that the responsible physician may consider the potential infectious risk associated with the vaccines and use short deferral periods (e.g. 14 days) for live attenuated vaccines and no deferral for non-replicating, inactivated, or RNA-based vaccines [5]. The FDA also agrees that no deferral is necessary for routine blood donors who received the mRNA-1273 or moderna vaccine [5]. On December 15^th^ 2020, in response to the AABB summary about donation of blood components following COVID-19 vaccines, FDA confirmed that it would be reasonable to have no donor deferral following mRNA COVID-19 vaccines and offered that a 14-day deferral is appropriate following a live-attenuated vaccine [6]. Also, any decision to update policies for deferral following COVID-19 vaccines is left to the discretion of the medical director [6]. On its updated report on December 23^rd^ 2020, AABB confirmed all recommendations cited previously and insisted on noting that a 12-month deferral is needed for recipients of unlicensed vaccines, subject to review by the medical director [7].

This review allows a medical director the discretion to define a deferral period of less than 12 months, such as those described above, based on information received from the FDA [7]. In the January 19^th^ 2021 notice, the FDA insisted on the same criteria already mentioned in its previous reports and reported that individuals uncertain about which COVID-19 vaccine was administered refrain from donating for a short waiting period (e.g. 14 days) [8]. On January 15^th^ 2021, the High Council of Public Health (HCPH) gave a provisional response on donors vaccinated with messenger RNA vaccines in use in France [1]. On February 2^nd^ 2021, the HCPH published a report relating to the exclusion criteria for donors of products from the human body who have been vaccinated against SARS-CoV-2 [1]. Considering the nature, modes of action of these vaccines, and the need for an adequate supply of blood and other human body products, the HCPH recommends no exclusion, even temporary, of donors who have received a messenger RNA and non-replicating vector vaccine authorized in the European Union (EU). The HCPH recommended the respect, as a precaution, for a temporary 4-week donation exclusion for vaccinated donors from countries outside the EU [1]. In addition, the HCPH insisted not to impose exclusion from donation for donors who have received vaccines based on protein subunits or viral pseudo-particles authorized in Europe due to the lack of risk for the recipient of this type of vaccine technology which is proven [1]. For all other vaccines (DNA vaccines, replicative vector vaccines, attenuated vaccines) which are not yet present on the European market, to reconvene the HCPH for specific recommendations if necessary [1]. The summary of all previous recommendations from international scientific societies is presented in Table 2.

**Table 2:** recommendations from international scientifics societies concerning the eligibility criteria for donating blood after COVID-19 vaccination

	Date of the report	Type of COVID-19 vaccine received	Recommendations	Other information's
ECDC	10^th^ December 2020	Inactivated vaccine	No postponement	Member states may consider taking a precautionary approach and defer donors who develop symptoms directly after receiving a SARS-CoV-2 vaccine for up to seven days after symptoms have resolved
		mRNA vaccine	No postponement	
		Vaccine with protein subunits	No postponement	
		Attenuated vaccine	Postponement of 04 weeks	
AABB	20^th^ November 2020	Non-replicating vaccine	No postponement	The FDA does not have a policy requiring the postponement of routine blood donors following COVID-19 vaccination. These FDA criteria were brought in at the request of the AABB
		Inactivated vaccine	No postponement	
		RNA vaccine	No postponement	
	12^th^ December 2020	mRNA vaccine	No postponement	The FDA does not have a policy requiring the postponement of routine blood donors following COVID-19 vaccination. It was at the request of the AABB that the FDA considered these recommendations
		Live attenuated vaccine	14 days deferral	
	23^th^ December 2020	Live attenuated vaccine	Postponement of 14 days from inoculation	
		Non-replicating vaccine	No postponement	
		Inactivated vaccine	No postponement	
		RNA-based vaccine (Moderna, Pfizer BioNTech)	No postponement	
		Vaccine without licence	12 month deferral	Subject to review by the medical director. This review gives a medical director the discretion to set a deferral period of less than 12 months, based on information received from the FDA
FDA	19^th^ January 2021	Non-replicating vaccine	No postponement	
		Inactivated vaccine	No postponement	
		RNA vaccine	No postponement	
		Live attenuated vaccine	14 days deferral	
		Donors who do not know the type of vaccine	14 days deferral	
HCPH	02^th^ February 2021	Vaccines with messenger RNA and non-replicating viral vectors (available now or in the near future in the European area)	No exclusion, even very temporary, is made for a blood donation	Not to impose any exclusion from the donation for donors who have received vaccines based on protein subunits or viral pseudo-particles, For all other vaccines (DNA vaccines, replicative vector vaccines, attenuated vaccines), to bring together SECPROCH again in order to establish specific recommendations if these vaccines were marketed in Europe, which does not this is not the case for the coming months; these recommendations, drawn up on the basis of the knowledge available on the date of publication of this opinion, may change depending on the updating of knowledge and epidemiological data

**Some recent experiences from blood transfusion establishments at the international level**: blood transfusion centres have already updated the eligibility criteria for blood donation after the COVID-19 vaccination. The American Red Cross (AmRC), following the FDA blood donation eligibility guidance, has updated its donor criteria eligibility and stipulates that deferral times may vary depending on the type of vaccine received [9]. For the AmRC, there is no deferral time if the blood donor receives the Pfizer or Moderna vaccine. If the blood donor received a COVID-19 vaccine, he would need to provide the manufacturer's name when he donated. The AmRC encourages donors to bring the COVID-19 vaccination card with them to their next donation [9]. Eligible blood donors who do not know what type of COVID-19 vaccine they received must wait four weeks before giving blood [9]. All donors must be symptom-free and feel well at the time of donation. If an individual is experiencing any symptoms from the COVID-19 vaccine, the red cross asks that they postpone their donation until they feel better [9]. The blood bank of Hawaii on December 8^th^ 2020, COVID-19 update, stipulated no deferral need after receiving Pfizer or Moderna COVID-19 vaccines [10]. The Scottish National Blood Transfusion Service on December 21^st^ 2020, stipulated that blood donors how had the vaccine as part of the UK vaccination program can give blood seven days after the jab [11]. The blood donor also has to be recovered from any reaction to the vaccine. If the blood donor has had a vaccine as part of a clinical trial, he might have to wait longer to donate [11]. The Singapore blood bank has implemented new guidelines [12]. For this blood bank, the deferral period may vary depending on the type of COVID-19 vaccine received or if the donor develops symptoms after receiving the vaccine [12]. Donors are required to provide information about their vaccination: date of vaccination, the brand of vaccine, as well as any side effects [12]. For inactivated viruses´ vaccines or vaccines containing no live agents (e.g. Pfizer-BioNTech vaccine), the donor is eligible if he doesn´t have developed any side effects three days after vaccination. If the donor expressed side effects excluding fever, muscle pain, joint pain, and rash, he must wait one week after resolving side effects [12], and if the donor had developed side effects such as fever, muscle pain, joint pain, and rash, he must wait four weeks after side effects resolve before donating blood. For vector-based virus or live attenuated virus, the donor must wait four weeks after vaccination in the absence of side effects [12]. According to the Singapore blood bank, the four-week waiting period for the live attenuated vaccine ensures patient safety [12]. The blood of a recently vaccinated donor may contain an infectious agent that can theoretically pose a risk to immunocompromised patients such as cancer patients [12]. For The Australian Red Cross (AsRC), as a precaution, donors need to wait for 7-days after receiving the COVID-19 vaccine before donating blood, plasma, or platelets [13]. This wait time applies to all COVID-19 vaccinations, regardless of which vaccine that the donor receives [13]. The AsRC needs all blood donors to feel fit and well when they give blood or plasma, and they don´t allow anyone with a fever to donate as part of their regular rules. Donors who donate when they´re not feeling 100 percent can be at an increased risk of experiencing an adverse reaction, such as fainting, during or after their donation [13].

On December 16^th^ 2020, the Canadian blood services, in its COVID-19 update, stipulated that for the COVID-19 vaccine, there is no deferral period post-vaccination [14]. India's National Blood Transfusion Council (NBTC) has recommended a donor deferral period of 28 days after vaccination against COVID-19 [15]. The Indian COVID-19 vaccination campaign includes Covishield (no replicative viral vector vaccine) and Covaxin (inactivated vaccine). Both have a two-dose regimen, in which doses are administered four weeks apart. Therefore, anyone undergoing the COVID-19 vaccine is effectively deferred for 28 days after the last dose [15]. Some experts criticize this decision in blood transfusion from India, who considers that the deferral of 28 days adopted by National Board Certified Teachers (NBCT) in India is just unjustified and unacceptable [15]. These Indian blood transfusion experts believe that the deferral period of 28 days from the last dose could result in a massive reduction in the number of eligible blood donors and compromise the blood supply management, which has already been disrupted during the COVID-19 pandemic [15]. They also report that there is no current evidence of the SARS-CoV-2 transfusion transmission, and that is still a theoretical risk [15]. The Indian blood transfusion experts propose a thorough revision of this decision to postpone donors for 28 days after COVID-19 vaccination, bearing in mind the already strained blood stocks at the respective blood transfusion services all over the nation [15].

**The Moroccan National Blood Transfusion Centre experience:** the Moroccan blood transfusion system includes one National Centre of Blood Transfusion and Hematology, 18 regional blood transfusion centres, 14 blood banks, and 24 blood transfusion units (antenna). The Moroccan National Centre for Blood Transfusion and Hematology (MNCBTH) is a scientific reference on a national scale. He is responsible for implementing the Moroccan Ministry of Health policy in the field of blood transfusion. Since the outbreak of the SARS-CoV-2 pandemic, he ensured health epidemiological and scientific watch to ensure good management of this health crisis. The MNCBTH has made several recommendations to provide, on the one hand, adequate technical support for regional blood transfusion centres and, on the other hand, the availability and safety of blood products [16]. The Moroccan COVID-19 vaccination campaign included Sinopharm (inactivated vaccine) and AstraZeneca (non-replicating viral vector vaccine). But other vaccine candidates could be considered in the Moroccan vaccination campaign against COVID-19 in the coming months. Considering the data available to date, the Moroccan National Centre for Blood Transfusion and Hematology adopted since the 6^th^ January 2021 new eligibility criteria for donating blood following COVID-19 vaccination. The MNCBTH advises that blood donors must provide all the necessary information about their vaccination: the date of vaccination, the type of vaccine received, and any side effects developed after the COVID-19 vaccination. All Moroccan Regional Blood Transfusion Centres and blood banks have to consider the new criteria adopted by the MNCBTH to determine eligibility for donating blood after vaccination against COVID-19 from the official launch of the COVID-19 vaccination campaign in Morocco in January 28^th^ 2021. The MNCBTH new eligibility criteria are presented in Table 3.

**Table 3:** the eligibility criteria adopted by the MNCBTH for blood donation after COVID-19 vaccination

Type of COVID-19 vaccine	Recommendations	
Inactivated vaccine mRNA-based vaccine Vaccine with protein sub-particles Non-replicative viral vaccine	No side effects after vaccination	Donation possible 07 days after the date of inoculation of the vaccine to the donor
	Development of side effects after vaccination	Donation possible 07 days after the date of declaration of complete resolution of symptoms
Live attenuated vaccine replicative viral vaccine	Postponement of 04 weeks	

## Conclusion

COVID-19 vaccines containing viral subunits, non-replicating viruses, inactivated viruses, or protein subunits are technologies that cannot be expected to include any element potentially harmful to a recipient of a blood product from a vaccinated donor. No exclusion for blood donation should apply to blood donors who have received this type of COVID-19 vaccine. Measures taken by some blood transfusion centres to observe a period of deferment of donors for 3 days or more in the event of secondary effects after COVID-19 vaccination are preventive measures based on the principle that blood donors must feel well in good health on the day of donation. COVID-19 live attenuated vaccines or replicative vectors vaccines are in the initial phase of clinical trials and not yet available for use. Those vaccines could justify a temporary exclusion of blood donors ranging from 2 to 4 weeks.

### What is known about this topic

- The medical screening practices for blood donor eligibility after vaccination against several conventional infectious agents are well known to blood transfusion centres worldwide;
- For classic infectious agents, blood donation is contraindicated four weeks after a live attenuated vaccine injection;
- Other types of vaccines (inactivated vaccines, antigens, or protein subunits) do not entail any risk of infection and do not justify any postponement.

### What this study adds

- Several candidate vaccines against SARS-CoV-2, responsible for the current COVID-19 pandemic, are produced using eight different technological platforms, including DNA and RNA platforms that have never been the source of vaccines yet marketed in humans;
- At this stage of information, we know that for COVID-19, no DNA-based vaccine, live attenuated vaccine, or replicative vector vaccine has yet been marketed in the world; the COVID-19 vaccines now available worldwide are not expected to pose any particular safety concerns for recipients of blood products from vaccinated subjects;
- COVID-19 live attenuated vaccines or replicative vectors vaccines could justify a blood donor deferral of up to one month; COVID-19 vaccines containing viral subunits, non-replicating viruses, inactivated viruses, or protein sub-units do not require blood donation deferral.
